# A Novel Position-Sensitive Linear Winding Silicon Drift Detector

**DOI:** 10.3390/mi15040518

**Published:** 2024-04-12

**Authors:** Tao Long, Jun Zhao, Bo Xiong, Xinqing Li, Minghua Tang, Zheng Li

**Affiliations:** 1 School of Materials Science and Engineering, Xiangtan University, Xiangtan 411105, China; 201610131314@smail.xtu.edu.cn (J.Z.); boxiong65190@163.com (B.X.); xqli1996zz@163.com (X.L.); tangminghua@xtu.edu.cn (M.T.); 2 School of Integrated Circuits, Ludong University, Yantai 264025, China

**Keywords:** linear winding silicon drift detectors, position-sensitive, self-bias, drift channel, electric field, electric potential, electron concentration

## Abstract

A novel position-sensitive linear winding silicon drift detector (LWSDD) was designed and simulated. On the frontside (anode side), the collecting anodes were set on both sides of the detector, and an S-shaped linear winding cathode strip was arranged in the middle, which can realize independent voltage division and reduce the complexity of external bias resistor chain compared with the traditional linear silicon drift detector. The detectors were arranged in a butterfly shape, which increased the effective area of the detectors and improved the collection efficiency. The linear winding silicon drift detector can obtain one-dimensional position information by measuring the drift time of electrons. The 2D position information of the incident particle is obtained from the anodes coordinates of the readout signal. One-dimensional analytically exact solutions of electric potential and field were obtained for the first time for the linear winding silicon drift detector. The simulation results show that the electric potential distribution inside the detector is uniform, and the “drift channel” inside the detector points to the collecting anodes on both sides, which proves the reasonable and feasible design of the linear winding silicon drift detector.

## 1. Introduction

Since its invention by Gatti and Rehak [1], the Silicon Drift Detector (SDD) has found extensive applications in various fields, including high-energy physics [2,3,4], astrophysics [5,6], light sources for synchronizer [7,8], medical imaging [9,10,11], elemental analysis [12,13,14], and scientific research [15,16,17]. The SDD shows exceptional qualities such as higher energy resolution, excellent position resolution, a high signal-to-noise ratio, a thin dead layer, and a rapid response time, and the device can be cooled via electrical methods. Additionally, it is compatible with the CMOS process, enabling the production of large-area and highly sensitive silicon detectors with intricate structures, remarkable consistency, and excellent performance.

**Figure 1:**
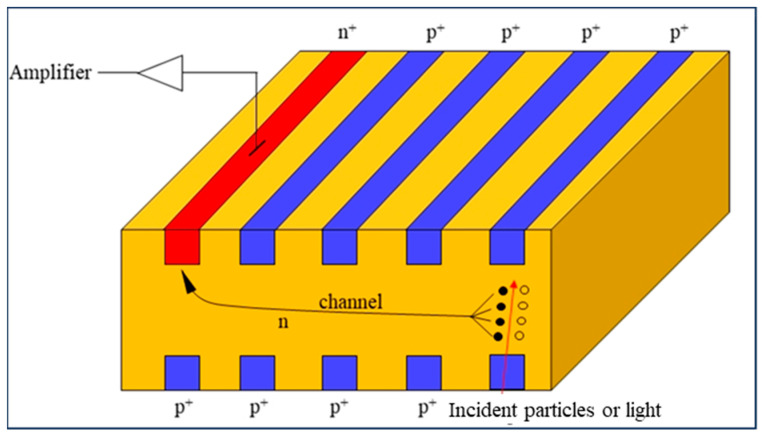
Schematic diagram of SDD.

The SDD operates on the idea of lateral depletion, where a high-resistivity silicon substrate has small n^+^-implanted anodes and a self-bias p^+^-implanted cathode on the frontside. By applying a bias voltage to the electrodes, the silicon substrate is fully depleted, creating a drift channel that is parallel to the surface. A p^+^-implanted cathode, which is identical to the one on the frontside, is equipped on the backside, or it covers the entire side as the electrode. The backside connections establish a certain voltage, creating a perpendicular electric field component relative to the surface of the detector. In the meantime, the backside serves as the incident surface of the detector. Figure 1 demonstrates that when X-rays or particles hit the depleted detector, an enormous number of electron–hole pairs are generated. The electrons travel towards the anode and are collected there, while the holes move towards the cathode.

A novel position-sensitive linear winding silicon drift detector (LWSDD) is proposed in this paper. The n^+^-implanted anodes of the detector are arranged on both sides of the detector, and the S-shaped linear microstrip p^+^-cathode chain is arranged in the middle, which can not only act as the field electrode but also a self-bias voltage divider. Compared with the traditional linear silicon drift detector [18,19], it simplifies the external bias voltage divider and simply requires a bias voltage differential at both ends of the strip electrode. The self-bias of the linear winding p^+^-cathode chain can be achieved over a wide area, leading to a full depletion of the detector. The detectors are arranged in a configuration that looks like a butterfly, therefore enhancing the general size of the detectors and optimizing the effectiveness of the particle collection. We created two designs for the n^+^-implanted anodes: a microstrip anode and a pixel anode. Assembling the n^+^-implanted anode in the form of a micro-strip allows one to obtain one-dimensional position resolution by evaluating the electron drift time. When configuring the n^+^-implanted anode as numerous small pixels collecting anode, the two-dimensional position information of the incident particles can be obtained from the anode coordinates by numbering the collecting anodes in the orderly direction.

## 2. Design of the LWSDD Structure

**Figure 2:**
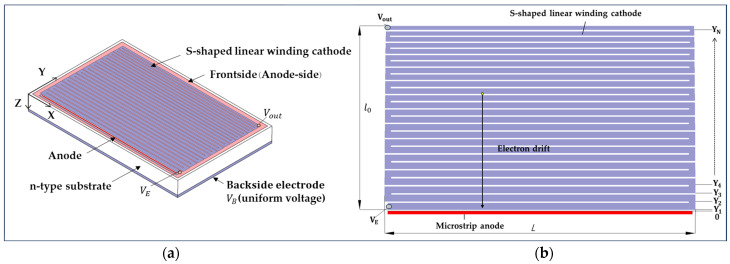
The linear winding silicon drift detector structure: (**a**) structural model of the LWSDD; (**b**) the frontside (anode side) configuration of the LWSDD.

The S-shaped linear winding frontside p^+^-implanted cathode and the backside p^+^-implanted cathode determine the potential distribution of the LWSDD. The frontside of the design features an S-shaped winding cathode chain, while the backside is p^+^-implanted over the entire surface, ensuring a consistent and uniform voltage. As depicted in Figure 2, we designate the x-direction as the length direction of the microstrip cathode, the y-direction as the direction of the microstrip cathode arrangement, and the z-direction as the thickness direction of the detector. Figure 2b depicts the frontside (anode side) configuration of the LWSDD. L is the length of the microstrip cathode, and $l_{0}$ is the width of microstrip winding cathode chain. Voltages of $V_{E}$ and $V_{out}$ are applied, respectively, on both ends of the S-shaped winding cathode chain (uniform voltage is applied on the backside, $V_{B}$). $Y_{1}$ is the distance from the first microstrip cathode to the collecting anode, whereas $Y_{N}$ is the distance from the final microstrip cathode to the collecting anode.

### 2.1. Surface Field Profile in LWSDD—An Analytically Exact Solutions

We first provide one-dimensional analytically exact solutions of the electric potential and field with a reasonable approximation. The electrode situated on the surface of the detector acts to provide the potential distribution at every position inside the detector. The fundamental aspect of establishing the electrode configuration involves the determination of the electric potential field and distribution on the surface of the detector. The Poisson equation for the LWSDD is given by

(1) $$\frac{\partial^{2}\phi (X,Y,Z)}{\partial X^{2}}+\frac{\partial^{2}\phi (X,Y,Z)}{\partial Y^{2}}+\frac{\partial^{2}\phi (X,Y,Z)}{\partial Z^{2}}=\frac{eN_{eff}}{\varepsilon_{0}\varepsilon }.$$

The potential of an arbitrary point detector internal *φ* (*x*, *y*, *z*) meets the following approximation conditions:

(2) $$\frac{\partial^{2}\phi (X,Y,Z)}{\partial Z^{2}}\gg \frac{\partial^{2}\phi (X,Y,Z)}{\partial Y^{2}}\;\mathrm{and}\;\frac{\partial \phi (X,Y,Z)}{\partial X}=0.$$

The Poisson equation can be approximated by

(3) $$\frac{\partial^{2}\phi (Y,Z)}{\partial Z^{2}}\approx \frac{eN_{eff}}{\varepsilon_{0}\varepsilon },$$

where $N_{eff}$ is the effective doping concentration of the LWSDD, ε is the dielectric constant of silicon, $\varepsilon_{0}$ is the dielectric constant of the vacuum, and the solution of Equation (3) is given by

(4) $$\phi \left(Y,Z\right)=\frac{2eN_{eff}}{\varepsilon_{0}\varepsilon }Z^{2}+AZ+B\;.$$

Here, *A* and *B* are independent of *Z*; however, they can be functions of *Y*. Then, Equation (4) can be written in the following general way:

(5) $$\phi (Y,Z)=V_{fd}(\frac{Z}{d}{)}^{2}+A(Y)Z+B(Y),$$

where $V_{fd}=eN_{eff}d^{2}/2\varepsilon_{0}\varepsilon$ is the full depletion voltage, and d is the thickness of the LWSDD. Φ(Y) and Ψ(Y) are the potential profiles of frontside (z=0) and backside (z=d), respectively:

(6) Φ(Y)=ϕ(Y,0) and Ψ(Y)=ϕ(Y,d).

Then, Equation (5) can be rewritten as

(7) $$\phi (Y,Z)=V_{fd}(\frac{Z}{d}{)}^{2}+(\frac{\Psi (Y)-\Phi (Y)-V_{fd}}{d})Z+\Phi (Y).$$

The partial derivative in the y direction of Equation (7) is applied to obtain the electric field component along the y direction of the internal electric field of the detector $E_{dr,Y}$:

(8) $$E_{dr,Y}(Y)=\frac{1}{2}\cdot (\frac{d\Psi (Y)}{dY}+\frac{d\Phi (Y)}{dY})-\frac{1}{2}\cdot \left[\frac{\Psi (Y)-\Phi (Y)}{V_{fd}}\right]\cdot \left[\frac{d\Psi (Y)}{dY}-\frac{d\Phi (Y)}{dY}\right].$$

The simplest approximation solution for the surface electric field $E_{dr,Y}\left(Y\right)=E_{dr,Y}=constant$, and a curved drift channel has been given in Ref. [20]. In this design, we set $E_{dr,Y}\left(Y\right)=E_{dr,Y}=constant$, as well as a curved drift channel.

Let us make the backside potential proportional to front side one:

(9) $$\Psi \left(Y\right)=V_{B}+\gamma \Phi (Y)\;(0\leq \gamma <1).$$

As indicated in Figure 2, the boundary conditions are set. The first cathode applied voltage is $\Phi \left(Y_{1}\right)=\left|V_{E}\right|$, the last cathode applied voltage is $\Phi \left(l_{0}\right)=\left|V_{out}\right|$, and a uniform voltage applied on the backside is $V_{B}$. Equation (8) can be transformed into

(10) $$E_{dr,Y}(Y)=\frac{1}{2}\frac{d\Phi (Y)}{dY}\left[1+\gamma +(1-\gamma )\frac{V_{B}-\left(1-\gamma \right)\Phi \left(Y\right)}{V_{fd}}\right]=constant=E_{dr,Y}.$$

We set

(11) $$\varphi (Y)=1+\gamma +\left(1-\gamma \right)\frac{V_{B}-\left(1-\gamma \right)\Phi \left(Y\right)}{V_{fd}}\;\;(\gamma \neq 1).$$

Adding the boundary conditions to Equation (11), we have

(12) $$\left\{\begin{array}{c} \varphi (Y_{1})=1+\gamma +(1-\gamma )\frac{V_{B}-\left(1-\gamma \right)V_{E}}{V_{fd}}\\ \varphi (l_{0})=1+\gamma +(1-\gamma )\frac{V_{B}-\left(1-\gamma \right)V_{out}}{V_{fd}} \end{array}\right..$$

Taking the derivative of Equation (11), we have

(13) $$d\varphi (Y)=-\frac{{\left(1-\gamma \right)}^{2}}{V_{fd}}d\Phi \left(Y\right).$$

By combining Equation (10) and Equation (13), we can obtain

(14) $${YE}_{dr}=-\frac{V_{fd}}{{4\left(1-\gamma \right)}^{2}}\varphi^{2}\left(Y\right)+C.$$

Adding the boundary conditions to Equation (14), we have

(15) $$C={Y_{1}E}_{dr}+\frac{V_{fd}}{{4\left(1-\gamma \right)}^{2}}\varphi^{2}\left(Y_{1}\right).$$

For different $E_{dr}(Y)$ we have

(16) $$\varphi^{2}\left(Y\right)=\frac{{4\left(1-\gamma \right)}^{2}}{V_{fd}}\left[C-\int E_{dr}(Y)dY\right].$$

If we choose $E_{dr}\left(Y\right)=E_{dr}{\left(\frac{Y}{Y_{1}}\right)}^{\eta }$, Equation (16) then becomes

(17) $$\varphi^{2}\left(Y\right)=\frac{{4\left(1-\gamma \right)}^{2}}{V_{fd}}\left[C-E_{dr}Y_{1}\frac{{\left(Y/Y_{1}\right)}^{\eta +1}}{\eta +1}\right].$$

Adding the boundary conditions to Equation (17), we have

(18) $$\left\{\begin{array}{c} \varphi^{2}\left(Y_{1}\right)=\frac{{4\left(1-\gamma \right)}^{2}}{V_{fd}}\left[C-\frac{E_{dr}Y_{1}}{\eta +1}\right]\\ \varphi^{2}\left(l_{0}\right)=\frac{{4\left(1-\gamma \right)}^{2}}{V_{fd}}\left[C-E_{dr}Y_{1}\frac{{\left(l_{0}/Y_{1}\right)}^{\eta +1}}{\eta +1}\right]\\ C=\frac{E_{dr}Y_{1}}{\eta +1}+\frac{V_{fd}}{{4\left(1-\gamma \right)}^{2}}\varphi^{2}\left(Y_{1}\right) \end{array}\right..$$

Based on the above calculation and solving Equation (10), as we expected, we obtain the constant drift field component in the *Y* direction ($E_{dr,Y}\left(Y\right)=E_{dr}$) and a curved drift channel:

(19) $$\;E_{dr}=\frac{\left(\eta +1)(V_{out}-V_{E}\right)}{4Y_{1}\left[{\left(l_{0}/Y_{1}\right)}^{\eta +1}-1\right]}\left\{2\left(1+\gamma \right)+\frac{1+\gamma }{V_{fd}}\left[2V_{B}-\left(1+\gamma \right)(V_{out}+V_{E})\right]\right\}=constant\;$$

(20) $$\mathrm{or}\;\;E_{dr}=\frac{(\eta +1)V_{fd}}{4Y_{1}{\left(1-\gamma \right)}^{2}\left[{\left(l_{0}/Y_{1}\right)}^{\eta +1}-1\right]}\left[\varphi^{2}\left(Y_{1}\right)-\varphi^{2}\left(l_{0}\right)\right]=constant.$$

The front surface electric potential and field are

(21) $$\Phi \left(Y\right)=\frac{1}{1-\gamma }\left\{V_{B}-\frac{V_{fd}}{1-\gamma }\left[\varphi \left(Y\right)-\left(1+\gamma \right)\right]\right\}$$

(22) $$E\left(Y\right)=\frac{d\Phi (Y)}{dY}=\frac{2E_{dr}\left(Y\right)}{\varphi (Y)}=\frac{2E_{dr}{\left(\frac{Y}{Y_{1}}\right)}^{\eta }}{\varphi (Y)}.$$

We need to avoid this singularity on the surface; hence, φ(Y)>0, and there is an upper limit to the values of $V_{B}$ and $V_{out}$:

(23) $$\left\{\begin{array}{c} V_{B}\leq V_{fd}\\ {\;V}_{out}\leq \frac{\left(1-\gamma \right)\left(1+\gamma \right)V_{B}+\left(1+\gamma \right)V_{fd}}{{\left(1-\gamma \right)}^{2}}\leq \frac{{2V}_{fd}}{{\left(1-\gamma \right)}^{2}} \end{array}\right..$$

We can obtain the electron drift time by calculating $t_{dr}$:

(24) $$t_{dr}=\int_{Y_{1}}^{Y}\frac{dY}{\mu E_{dr}(Y)}=\frac{Y_{1}}{\mu E_{dr\left(1-\eta \right)}}\left[{\left(\frac{Y}{Y_{1}}\right)}^{1-\eta }-1\right].$$

### 2.2. Linear Winding Electrode Design

**Figure 3:**
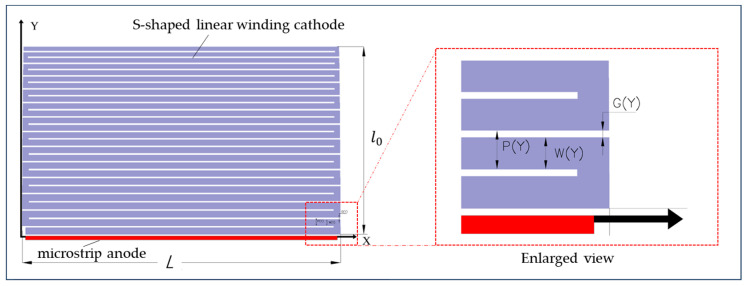
The layout of electrodes for a microstrip anode LWSDD.

In general, the surface potential and field profiles we calculated above can be used to design the LWSDD. Figure 3 displays the design diagram of the electrode configuration on the front side of the microstrip anode LWSDD. The red portion represents the n^+^-implant microstrip anode, and the purple portion shows the p^+^-implant S-shaped linear winding cathode. L is the length of one sector of the microstrip winding cathode, set as the X coordinate, and $l_{0}$ is the width of microstrip cathode chain, set as the Y coordinate. We assign specific values for the parameters calculated above. The detector has a thickness of 500 μm (d=500 μm), and the substrate doping concentration is 4 × 10^11^/cm^3^; the microstrip anode (the red portion) was doped with phosphorus at a concentration of 1 × 10^19^/cm^3^. The linear winding cathode chain (the purple portion) was doped with phosphorus at a concentration of 1 × 10^19^/cm^3^. The microstrip cathode length L=3200 μm, and the microstrip cathode chain width $l_{0}=2000$ μm. The boundary conditions of the applied voltage are: $V_{E}=-10\;V$, $V_{out}=-100\;V$, and uniform voltage is applied on the backside $V_{B}=-76\;V$ (γ=0); η=−0.33. As shown in Figure 3, an enlarged view, $P\left(Y\right)$ is the microstrip winding cathode pitch, $W\left(Y\right)$ is the width of the microstrip winding cathode, G(Y) is the gap of the microstrip winding cathode, and the relationship among the three is as follows: $P\left(Y\right)=W\left(Y\right)+G(Y)$.

In this design, G(Y) is set as a constant, and G=15 μm; hence, the relation is

(25) $$P\left(Y\right)=W\left(Y\right)+G.$$

The total length of the microstrip winding cathode chain is

(26) $$l_{C}=NG+(N+1)L.$$

The total resistance of the microstrip cathode chain is

(27) $$Rc=\sum_{i=1}^{N}\frac{\rho_{s}\left[L+G_{i}\left(Y\right)\right]}{W_{i}\left(Y\right)}+\frac{\rho_{s}L}{W_{n+1}\left(Y\right)}\;,$$

where $\rho_{s}$ is the block resistivity of the resistance of the ion implanted cathode. The voltage drop between two adjacent cathodes is

(28) ΔV=IR=E(Y)P(Y).

It can be shown that the pitch $P\left(Y\right)$, gap G, front surface field $E\left(Y\right)$, implant sheet resistance $\rho_{s}$, current I, and length of the cathode are related as follows:

(29) $$\left(L+G\right)I\rho_{s}=E\left(Y\right)P(Y)\left[P\left(Y\right)-G\right].$$

Then, we can obtain:

(30) $$P\left(Y\right)=\frac{1}{2}\left[G+\sqrt{G^{2}+4I\rho_{s}\left(L+G\right)/E\left(Y\right)}\right].$$

**Figure 4:**
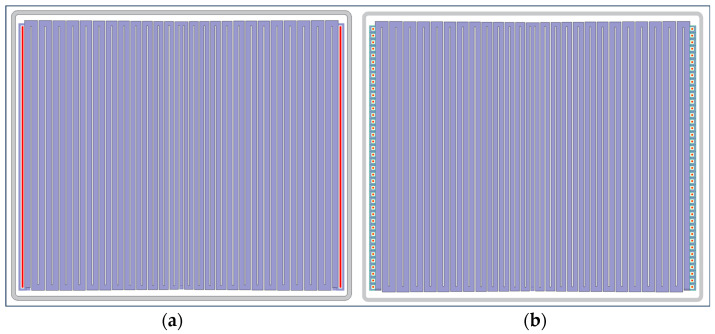
Design layout of the LWSDD: (**a**) design layout of the microstrip anodes LWSDD; (**b**) design layout of the pixel anodes LWSDD.

By completing the aforementioned calculation, we acquired all the necessary parameter values and subsequently devised two types of LWSDD: microstrip anodes LWSDD and pixel anodes LWSDD, as depicted in Figure 4. The detectors are arranged in a butterfly shape, which increases the effective area of the detectors and improves the collection efficiency. The LWSDD can obtain one-dimensional position information by measuring the drift time of electrons. The 2D position information of the incident particle is obtained from the anode coordinates of the readout signal.

## 3. Modeling and 3D TCAD Simulation of LWSDD

**Figure 5:**
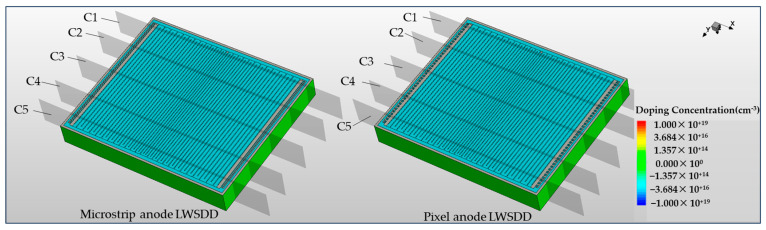
Cutplanes along the Y axis: C1 is at Y = 120 μm; C2 is at Y = 800 μm; C3 is at Y = 1600 μm; C4 is at Y = 2400 μm; C5 is at Y = 3000 μm.

We employed Sentaurus TCAD to 3D simulate the LWSDD to increase the accuracy of investigating the electrical characteristics of the detector by incorporating the full 3D boundary conditions of the detector. The detector substrate consisted of n-type silicon, measuring 500 μm in thickness and having a doping concentration of 4 × 10^11^/cm^3^. We doped the anodes with phosphorus at a concentration of 1 × 10^19^/cm^3^. We doped the S-shaped winding cathode chain with boron at a concentration of 1 × 10^19^/cm^3^. The detector had a symmetrical arrangement in a butterfly configuration, forming an effective area of 3200 μm × 4000 μm. The detector utilized a reverse bias voltage to deplete the substrate. The initial cathode adjacent to the anode had a voltage $V_{E}=-10\;V$, whereas the final cathode had a voltage $V_{out}=-100\;V$. The backside was a whole electrode, and it had a voltage $V_{B}=-76\;V$. Using the calculated data $P\left(Y\right)$, $W\left(Y\right)$, and the specified parameter values of L, $l_{0}$, and G, we configured the 3D modeling, as shown in Figure 5. In order to better examine the electric field, electric potential, and electron concentration distribution within the detector, we made cuts along the *Y* axis at specific points: 120 μm, 800 μm, 1600 μm, 2400 μm, and 3000 μm. We made these cuts within the detector’s large effective area. Subsequently, we analyzed the results of the simulation.

### 3.1. Electric Field Distribution of LWSDD

**Figure 6:**
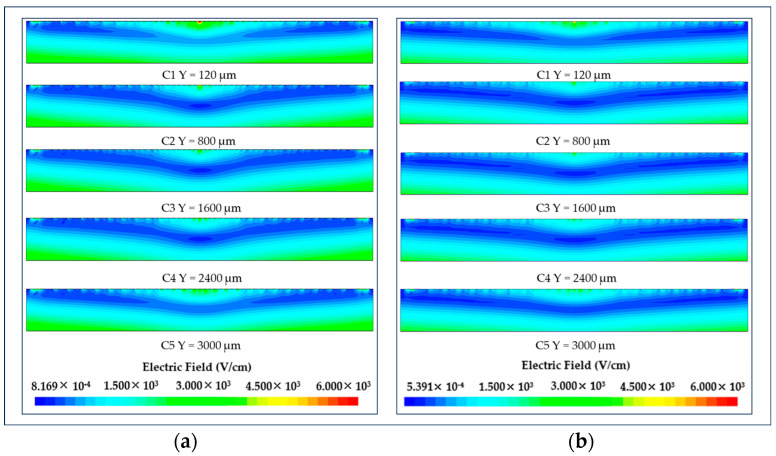
Electric field distribution in different cutplanes along the Y axis: (**a**) electric field distribution in microstrip anode LWSDD; (**b**) electric field distribution in pixel anode LWSDD.

Figure 6 demonstrates that the electric field of the LWSDD, which includes microstrip and pixel anodes, exhibits a symmetrical distribution. This aligns with our design goal, indicating that the detectors on both sides have equal effectiveness at the level of performance and effectively increasing the detection area. Within the detector, there exists a zone that features a weak electric field that extends diagonally from the central position of the detector to the location of the collecting anodes on both sides. The electron drift channel lies in this area of low electric field, which implies that the drift field is much smaller than the confining field.

**Figure 7:**
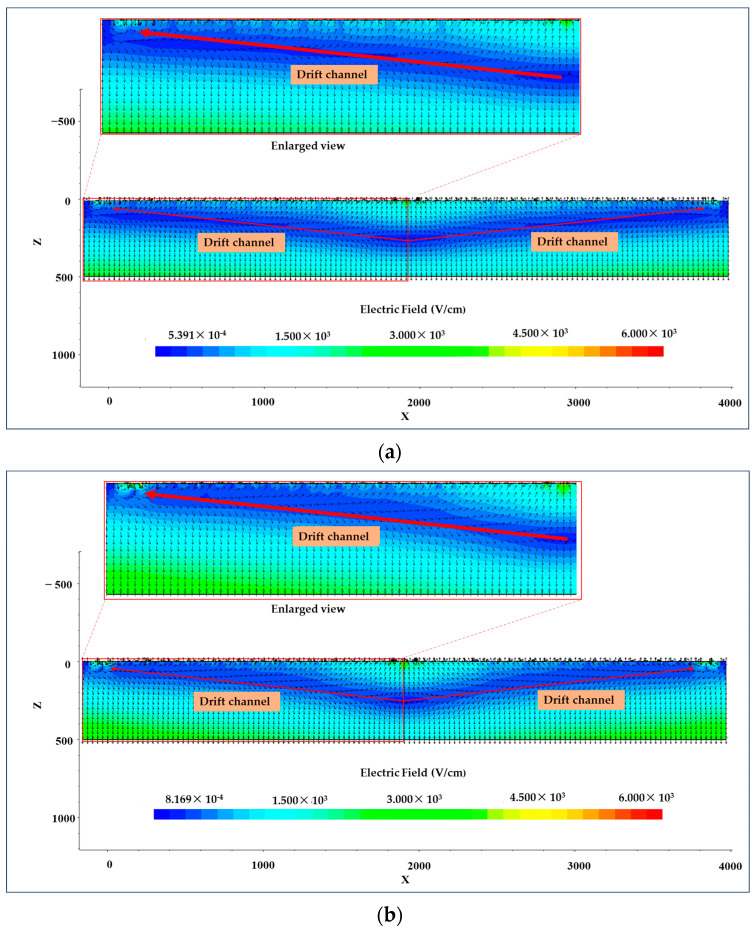
Vector electric field at Y = 1600 μm: (**a**) vector electric field in microstrip anodes LWSDD; (**b**) vector electric field in pixel anodes LWSDD.

Figure 7 shows the distribution of vector electric field at Y = 1600 μm. The direction opposite to that of the electric field vector arrow in the illustration corresponds to the direction of the electron drift. Regardless of the impacted particle’s initial position inside the effective area, its generated electrons will ultimately move towards the anodes on both ends and be collected there.

### 3.2. Potential Distribution of LWSDD

**Figure 8:**
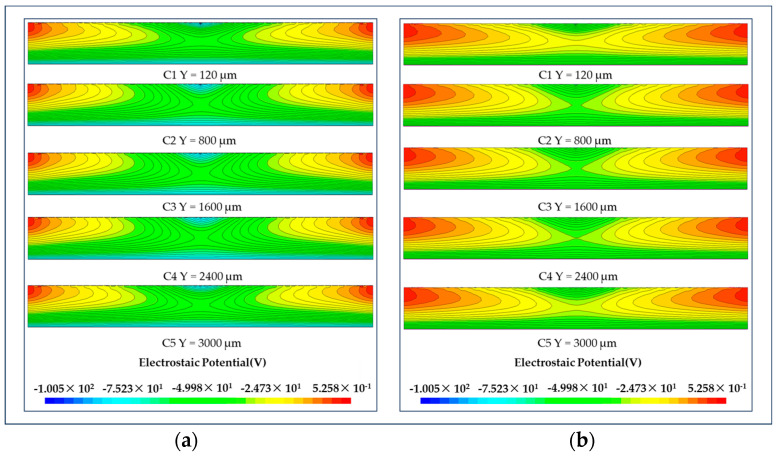
Electric potential distribution in different cutplanes along the Y axis: (**a**) electric potential distribution in microstrip anodes LWSDD; (**b**) electric potential distribution in pixel anodes LWSDD.

Figure 8 clearly shows that the two detectors’ electric potential are symmetrically distributed. The electric potential reaches its maximum value in the vicinity of the collecting anodes on both sides. In the Z direction (thickness direction), the electric potential consistently declines. Similarly, in the X direction, the electric potential steadily diminishes towards the middle of the detector. The shift in electric potential gradient supports the movement of electrons towards a collecting point, and it is evident that the electric potential gradient is highly uniform, resulting in a homogeneous electric field for the electron’s transport.

**Figure 9:**
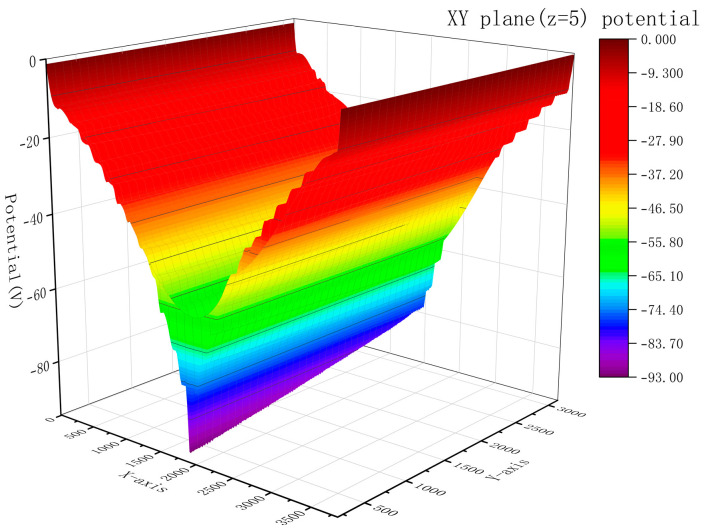
Electric potential distribution of the microstrip anodes LWSDD in the XY plane at Z = 5 μm.

To enhance the examination of the electric potential distribution inside the detector’s effective area, we captured the electric potential distribution in the XY plane at Z = 5 for the purpose of creating a 3D representation, as shown in Figure 9. The electric potential distribution along the Y direction of the detector is highly harmonious, which indicates that the electric potential distribution along the Y axis inside the detector is uniform. Particles entering any position within the effective area of the detector will be collected.

### 3.3. Electron Concentration Distribution of LWSDD

**Figure 10:**
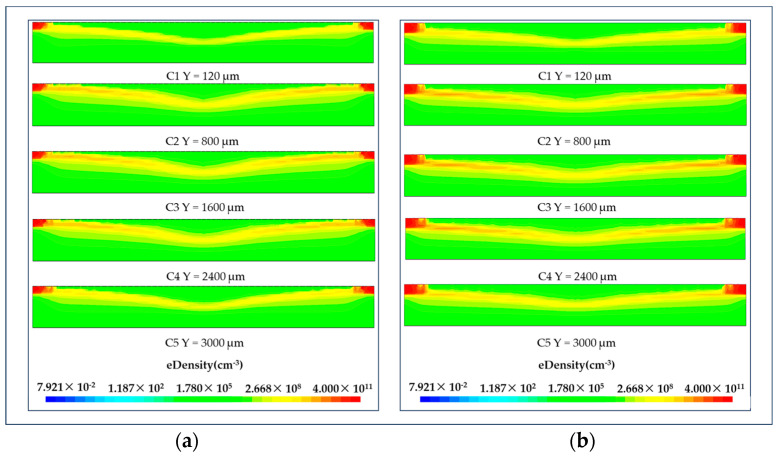
Electron concentration in different cutplanes along the Y axis: (**a**) electron concentration in microstrip anode LWSDD; (**b**) electron concentration in pixel anode LWSDD.

Referring to Figure 10, the electron concentration depicted here effectively visualizes the electron drift channels of the two detectors. The electron concentration distribution of the various sections in the figure is essentially the same, demonstrating a straight line from the detector’s middle position to the anode position on both sides, demonstrating the excellent and consistent performance of each position within the detector’s effective area. The figure illustrates that the maximum concentration in the drift channel path is approximately 10^9^, which is two orders of magnitude lower than the detector substrate concentration. This indicates that the winding cathode strip is effectively self-biased and fully depletes the detector by applying a bias voltage at both ends of the S-shaped winding cathode.

## 4. Conclusions

The work shows the design of a novel position-sensitive linear winding silicon drift detector. The collecting anodes of the detector are arranged on both sides of the detector. In the center, a linear winding microstrip cathode with an S-shaped configuration achieves a self-bias voltage divider, thus simplifying the external voltage divider in comparison to the conventional linear silicon drift chamber. Setting up the detector symmetrically in a butterfly shape effectively increases the detection area and improves the collection efficiency. By evaluating the drift time of the electrons, the linear winding silicon drift detector has the capacity to obtain one-dimensional position information. The readout signal’s anode coordinates determine the 2D position of the incident particle. The results that were obtained from the three-dimensional TCAD simulation of the detector demonstrate that the electric field distribution is evenly distributed and that the distribution of the electric potential in the effective area of the detector is consistent. It forms a drift channel near a straight line by applying a uniform electric field of approximately 200 V/cm, which can guarantee that the detector gathers electrons generated by incident particles more quickly and effectively. The design of the detector is confirmed to be reasonable and feasible, serving as a model for the development of linear winding silicon drift detector.

## Data Availability

The data presented in this study are available from the corresponding author upon reasonable request.
